# Pancreatic groove cancer

**DOI:** 10.1097/MD.0000000000005640

**Published:** 2017-01-13

**Authors:** Yuan-Hao Ku, Shih-Chin Chen, Bor-Uei Shyr, Rheun-Chuan Lee, Yi-Ming Shyr, Shin-E. Wang

**Affiliations:** a Department of Surgery; b Department of Radiology, Taipei Veterans General Hospital and National Yang Ming University, Taipei, Taiwan.

**Keywords:** groove cancer, groove pancreatitis, pancreatic

## Abstract

Pancreatic groove cancer is very rare and can be indistinguishable from groove pancreatitis. This study is to clarify the characteristics, clinical features, managements, and survival outcomes of this rare tumor.

Brief descriptions were made for each case of pancreatic groove cancer encountered at our institute. Individualized data of pancreatic groove cancer cases described in the literature were extracted and added to our database to expand the study sample size for a more complete analysis.

A total of 33 patients with pancreatic groove cancer were included for analysis, including 4 cases from our institute. The median tumor size was 2.7 cm. The most common symptom was nausea or vomiting (89%), followed by jaundice (67%). Duodenal stenosis was noted by endoscopy in 96% of patients. The histopathological examination revealed well differentiated tumor in 43%. Perineural invasion was noted in 90%, and lymphovascular invasion and lymph node involvement in 83%. Overall 1-year survival rate was 93.3%, and 3- or 5-year survival rate was 62.2%, with a median survival of 11.0 months. Survival outcome for the well-differentiated tumors was better than those of the moderate/poorly differentiated ones.

Early involvement of duodenum causing vomiting is often the initial presentation, but obstructive jaundice does not always happen until the disease progresses. Tumor differentiation is a prognostic factor for survival outcome. The possibility of pancreatic groove cancer should be carefully excluded before making the diagnosis of groove pancreatitis for any questionable case.

## 1 Introduction

Pancreatic groove cancer refers to the pancreatic cancer located in an anatomic area between the head of the pancreas, the duodenum and the common bile duct.^[1–3]^ Radiography often shows a plate-like mass for this disease, and histopathology reveals duodenal invasion which may cause duodenal stricture in the advanced-stage of the disease.^[2–4]^ The most common symptoms of pancreatic groove cancer can be abdominal pain and vomiting due to the duodenal stenosis.^[5,6]^ Most of the reported cases present clinical pictures similar to those seen in groove pancreatitis which is a more common clinical entity.^[2]^ Because of its rarity and limited clinical experiences, differentiation of pancreatic cancer from groove pancreatitis might be difficult in clinical diagnosis, and, moreover, groove pancreatitis can sometimes coexist with pancreatic cancer or may even mask the underlying problem.^[1,5,7–9]^The definitive diagnosis is frequently only obtained by histopathological examination after surgical intervention.^[1]^

The purposes of this article are to present our clinical experience with groove pancreatic cancer and to analyze an expanded sample size by adding cases from the literature to our pool of study cases. Thus, an attempt is made to clarify the characteristics, clinical features, managements, and survival outcomes of this rare tumor.

## 2 Methods

Brief descriptions were made for each case of pancreatic groove cancer encountered at our institute between 2012 and 2016. This study was approved by our institutional review board, and appropriate informed consent was obtained for each patient. To clarify the characteristics of pancreatic groove cancer, individualized data of pancreatic groove cancer cases described in the literature were extracted and added to our database to expand the study sample size for a more complete analysis. Two methods were utilized to search for relevant cases in the literature. First, to identify the relevant articles dealing with pancreatic groove cancer in the literature, a computerized search was performed through the PubMed electronic database, covering data from 2003 to 2015. The following keywords were used for the PubMed search: pancreas groove cancer, groove cancer, and pancreas groove adenocarcinoma. Second, the reference lists of PubMed-selected pancreas groove cancer articles were screened systematically for additional studies of interest. A total of 10 related articles were selected for the study.^[1–5,7,9–12]^ Cases without individualized data and duplicate cases reported in literature were excluded from the analysis. The data pool from the related literature and our patients’ cases were analyzed to determine the characteristics of pancreatic groove cancer including demographics, tumor size, clinical presentations, histopathology, treatment, and survival outcomes.

Statistical analysis was performed using SPSS 21.0 software (Statistic Package for Social Sciences, SPSS, Inc., Chicago, IL). All continuous data were calculated using median and mean ± standard deviation (SD), and frequency, as appropriate to the type of data. Actuarial survival was estimated via the Kaplan–Meier method, and a log rank test was used to determine difference in the subgroups. *P* < 0.05 was considered statistically significant.

## 3 Results

**Figure 1:**
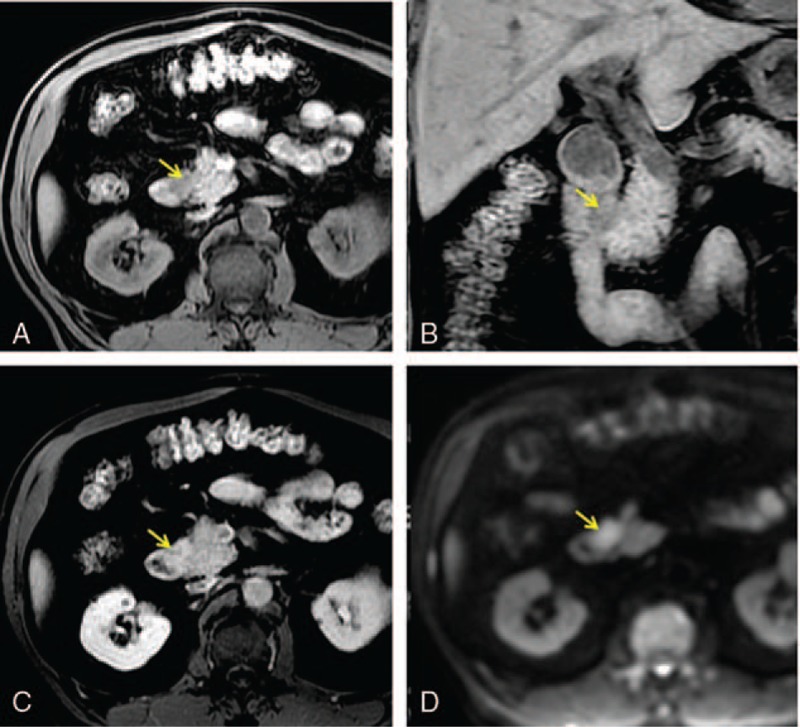
Axial (A) and coronal (B) T1-weighted fat-suppressed gradient-recalled echo MR image reveals a 2.0-cm hypointense lesion (arrow) in pancreatic groove. (C) The tumor becomes isointense in late arterial phase after contrast administration. (D) Diffusion-weighted MR image (b = 800 s/mm^2^) shows hyperintensity suggesting restricted diffusion.

There were 4 cases of pancreatic groove cancer in our institute, and 3 of them presented nausea/vomiting due to duodenal obstruction (Table 1). One patient was asymptomatic, with merely an elevation of serum carbohydrate antigen 19-9 (CA 19-9) at initial presentation, which was later diagnosed with pancreatic groove cancer by magnetic resonance imaging (MRI) (Fig. 1). Jaundice occurred in 1 of our 4 cases. Duodenal mucosa was examined with normal appearance by endoscopic examination or gross examination during operation in 3 patients. Perineural invasion was noted in all of our 4 cases, and lymphovascular invasion and lymph node involvement in 3.

**Table 1:**
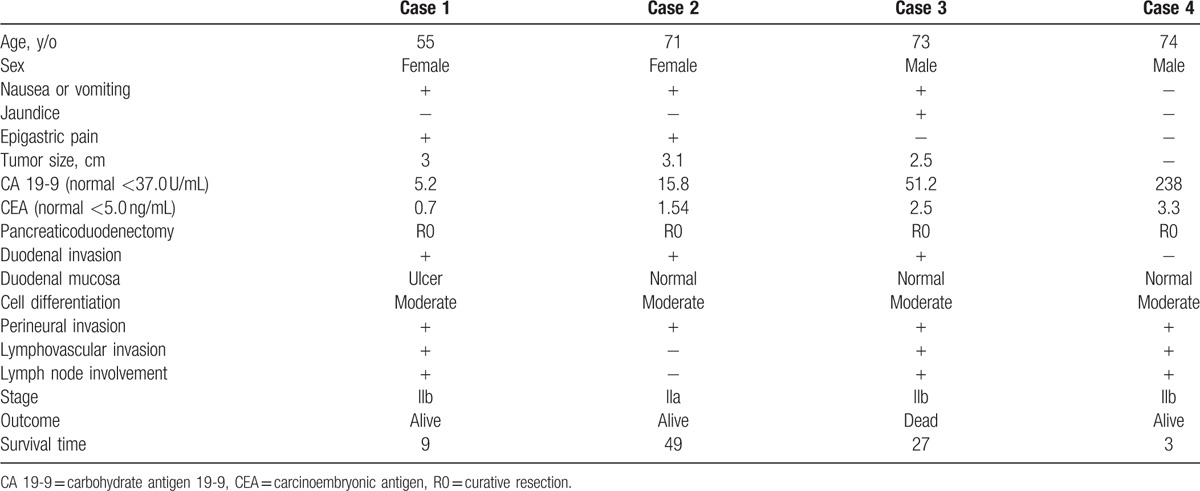
Pancreatic groove cancer from Taipei Veterans General Hospital.

A total of 33 patients with pancreatic groove cancer were included for analysis after adding cases from the literature to our pool of study cases (Table 2). It could occur between the ages of 26 and 83 years old, with a median of 69 years old. The median size of tumor was 2.7 cm, with a range from 2 to 3 cm. The most common symptom was nausea or vomiting (89%), followed by jaundice (67%). Only 18% patients were reported to have alcohol abuse. Duodenal stenosis was noted by endoscopy in 96% of patients, but 38% presented normal appearance of duodenal mucosa and 48% edematous mucosa under endoscopic findings. Elevation of serum CA 19-9 was noted in 60%.

**Table 2:**
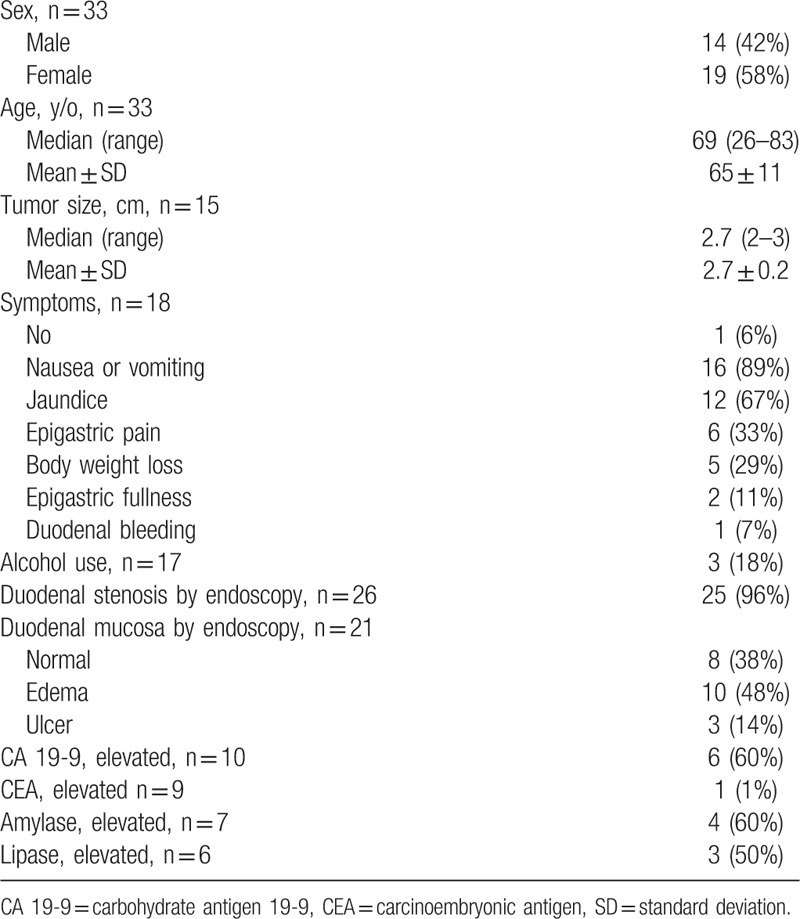
Demographics and clinical presentations of pancreatic groove cancer.

Malignancy was proved by endoscopic biopsy for pancreatic groove cancer in 58% and by intraoperative biopsy in 50%. Resections of the pancreatic groove cancer with pancreaticoduodenectomy were achieved in 76%, and 24% underwent bypass surgery. The histopathological examination revealed well differentiation of the tumor cells in 43% and moderate/poor in 58%. Perineural invasion was noted in 90%, and lymphovascular invasion and lymph node involvement in 83% (Table 3).

**Table 3:**
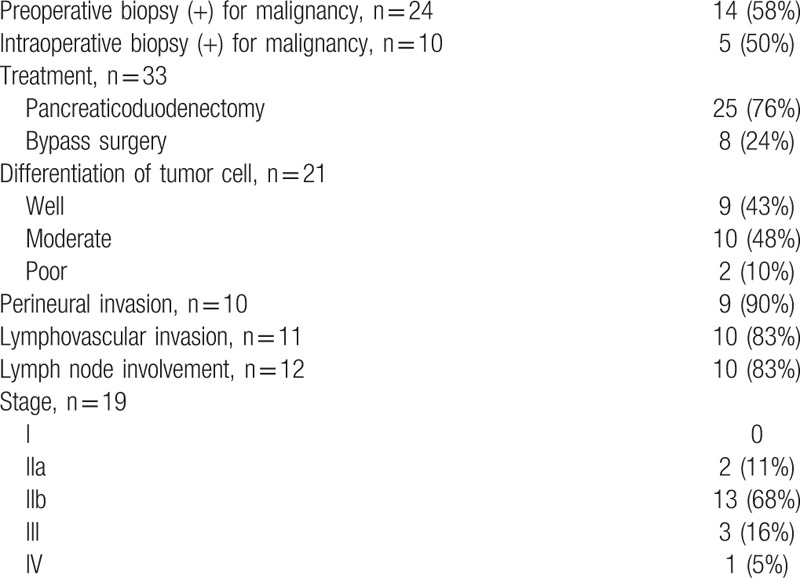
Pathology and treatment of pancreatic groove cancer.

**Figure 2:**
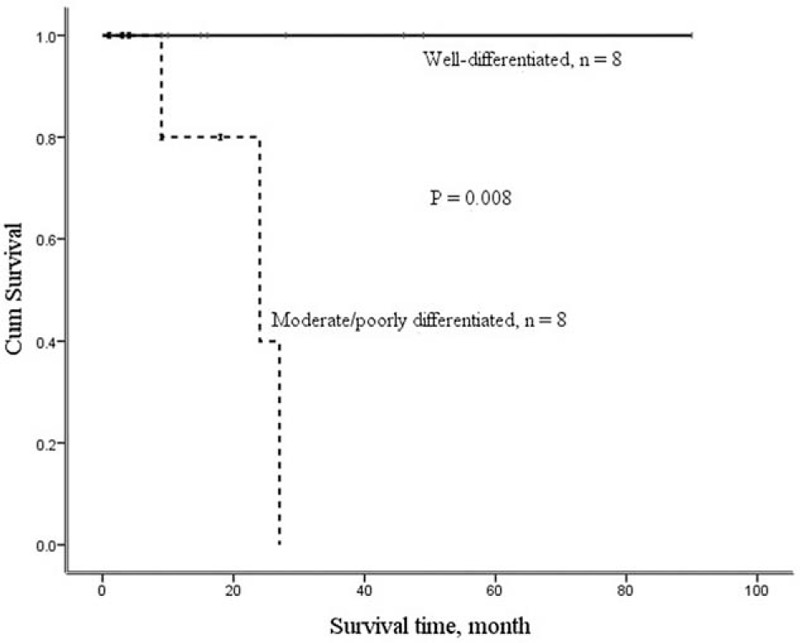
The cumulative survival curves for well-differentiated and moderate/poorly differentiated pancreatic groove cancers.

Overall 1-year survival rate was 93.3%, and 3- or 5-year survival rate was 62.2% for pancreatic groove cancer, with a median survival of 11.0 months (Table 4). Based on the tumor cell differentiation, survival outcome for the well-differentiated group was better than that for moderate/poorly differentiated group (Fig. 2).

**Table 4:**
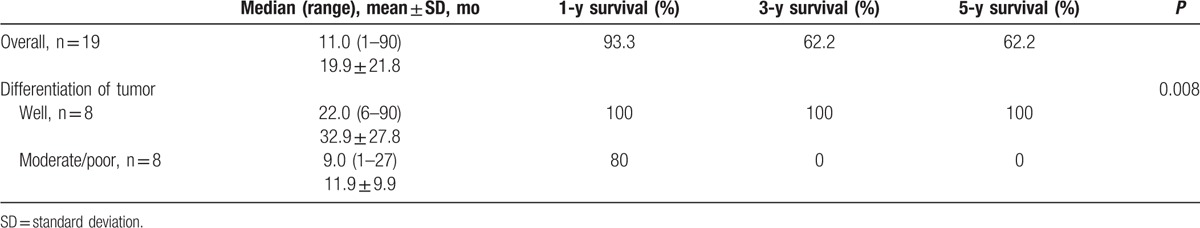
Survival outcomes of pancreatic groove cancer.

## 4 Discussion

Pancreatic groove area usually lacks pancreatic tissues and is mainly composed of fibrous connective tissues, small vessels, and nerves. However, a small amount of pancreatic tissue occasionally appear at the groove area.^[4,13]^ Therefore, either cancer or pancreatitis can occur in the pancreatic groove, although both of them are rarely seen clinically.

Like the usual pancreatic ductal adenocarcinoma, pancreatic groove cancer also appears in the sixth or seventh decade with less incidence of alcohol abuse.^[5]^ The median age for patients with this disease was 69 years and only 18% had alcohol abuse by our study. With the anatomic location between the duodenum and the common bile duct, pancreatic groove cancer easily exhibits not only distal common bile duct involvement but also duodenal invasion. It has been claimed that duodenal obstruction with nausea/vomiting is often an initial presenting symptom for most cases, but obstructive jaundice does not always happen until the disease progresses.^[2,4,5]^ The intrapancreatic common bile duct is only involved at the advanced stage.^[2]^ Three out of 4 patients in our study presented nausea/vomiting, with only 1 showing signs of jaundice. Our study also shows the most common presenting symptom is nausea/vomiting (89%), instead of jaundice (67%). In contrast, duodenal obstruction in periampullary cancer is often a late presentation and sometimes means a preterminal event at the advanced stage that causes nausea, vomiting, and ultimately cachexia^[2]^ symptoms.

Radiologically, T1-weighted and fat-suppressed T1-weighted MRI could show hypointensity, and T2-weighted images could be hyper- or isointensity. Dynamic computerized tomography (CT) and MRI could reveal hypovascularity in the early phase and delayed enhancement in the late phase of the lesion. However, these imaging findings can also be revealed in groove pancreatitis. The distinction between pancreatic groove cancer and fibrous scar in groove pancreatitis is often difficult on CT and MRI studies.^[3,9]^

Aimoto et al^[2]^ claimed that pancreatic groove cancer could infiltrate the duodenal submucosal layer histopathologically with circumferential pattern. Cancer cells could be found only in the mucosal layer at the early stage, and common bile duct could only be involved at the advanced stage. The feature of invasion of pancreatic groove cancer into the submucosal layer around the wall might explain that although duodenal stenosis was noted in 96% of patients, 38% of the duodenal mucosa could still look normal. Malignancy could be preoperatively confirmed by endoscopic biopsy in 58%. Our findings seemed to be in agreement with Aimoto report. Therefore, pathological findings of endoscopic biopsy specimens might show no evidence of malignancy at the early stage.^[2]^

It is crucial but challenging to differentiate pancreatic groove cancer from groove pancreatitis. Usually groove pancreatitis can be treated by conservative measures, and surgical resection should be reserved for patients with severe symptoms or in order to rule out malignancy. Similar to pancreatic groove cancer, patients with groove pancreatitis also frequently present with vomiting due to duodenal stenosis and obstructive jaundice due to common bile duct stenosis.^[2,3]^ Nevertheless, some features might tell the difference. Groove pancreatitis appears as a sheet-like mass, whereas pancreatic cancer manifests as an irregular mass. Stenosis of the distal common bile duct is smooth and long in groove pancreatitis, but abrupt and short in pancreatic cancer. Cystic dystrophy of the duodenal wall is more commonly seen in the duodenal wall in groove pancreatitis than in that of pancreatic cancer. However, arteries in pancreatic head lesions are often encased in pancreatic cancer but rarely in groove pancreatitis.^[3,14]^ Nevertheless, it is vital to include the differential diagnosis of pancreatic groove cancer in any questionable case, before making the diagnosis of groove pancreatitis. The possibility of pancreatic groove cancer should be carefully excluded.^[1]^

Although a very high positive rate of perineural invasion (90%), lymphovascular invasion (83%), and lymph node involvement (83%) for pancreatic groove cancer is noted in this study, the overall 1-year survival is 93.3% and 5-year survival 62.2%. However, with limited case number and short follow-up period, it is still hard to conclude that pancreatic groove cancer is better than that of pancreatic ductal adenocarcinoma. Tumor differentiation is a prognostic factor, and the well-differentiated pancreatic groove cancer can be associated with a favorable survival outcome, with 100% 1- and 5-year survival rates.

In conclusion, nausea/vomiting resulting from early involvement of duodenum is often an initial presenting symptom for most cases, but obstructive jaundice is not always inevitable until the disease progresses. Well-differentiated pancreatic groove cancer is associated with favorable survival outcome. Nevertheless, more cases and longer follow-up period are needed to confirm this before solid conclusion is made. Since it is often difficult to differentiate the pancreatic groove cancer from groove pancreatitis on either clinical features or imaging studies, the possibility of pancreatic groove cancer should be carefully excluded before making the diagnosis of groove pancreatitis for any questionable case.

## References

[1] Malde DJ Oliveira-Cunha M Smith AM Pancreatic carcinoma masquerading as groove pancreatitis: case report and review of literature. JOP 2011;12:598–602. 22072250

[2] Aimoto T Uchida E Nakamura Y Clinicopathologic study on pancreatic groove carcinoma. Pancreas 2006;33:255–9. 17003647 10.1097/01.mpa.0000236723.41919.90

[3] Gabata T Kadoya M Terayama N Groove pancreatic carcinomas: radiological and pathological findings. Eur Radiol 2003;13:1679–84. 12835985 10.1007/s00330-002-1743-1

[4] Izumi S Nakamura S Mano S Well differentiation and intact Smad4 expression are specific features of groove pancreatic ductal adenocarcinomas. Pancreas 2015;44:394–400. 25426619 10.1097/MPA.0000000000000260

[5] Goransky J Alvarez FA Picco P Groove pancreatitis vs groove pancreatic adenocarcinoma. Report of two cases and review of the literature. Acta Gastroenterol Latinoam 2013;43:248–53. 24303693

[6] Funamizu N Aramaki M Matsumoto T Groove pancreatic carcinoma. Hepatogastroenterology 2009;56:1742–4. 20214229

[7] Ishigami K Tajima T Nishie A Differential diagnosis of groove pancreatic carcinomas vs. groove pancreatitis: usefulness of the portal venous phase. Eur J Radiol 2010;74:e95–100. 19450943 10.1016/j.ejrad.2009.04.026

[8] Yamaguchi K Tanaka M Groove pancreatitis masquerading as pancreatic carcinoma. Am J Surg 1992;163:312–6. discussion 317–318. 1539765 10.1016/0002-9610(92)90009-g

[9] Tan CH Chow PK Thng CH Pancreatic adenocarcinoma that mimics groove pancreatitis: case report of a diagnostic dilemma. Dig Dis Sci 2006;51:1294–6. 16944029 10.1007/s10620-006-8052-5

[10] Badia Bartolome C Diaz Formoso FJ Rodriguez Falcon R Groove pancreatitis and its differential diagnosis with pancreatic adenocarcinoma. Gastroenterol Hepatol 2009;32:22–8. 19174095 10.1016/j.gastrohep.2008.09.005

[11] Meguro T Yamamoto T Nishioka K Pancreatic groove carcinoma in a young adult. Nihon Shokakibyo Gakkai Zasshi 2008;105:1078–86. 18603854

[12] Kuwatani M Kawakami H Yamato H Three cases of groove pancreatic carcinoma in which endoscopic ultrasonography was useful for diagnosis before surgery. Nihon Shokakibyo Gakkai Zasshi 2008;105:1061–9. 18603852

[13] Suda K Histopathology of the minor duodenal papilla. Dig Surg 2010;27:137–9. 20551659 10.1159/000286920

[14] Tezuka K Makino T Hirai I Groove pancreatitis. Dig Surg 2010;27:149–52. 20551662 10.1159/000289099

